# Pediatric 320-row cardiac computed tomography using electrocardiogram-gated model-based full iterative reconstruction

**DOI:** 10.1007/s00247-017-3901-2

**Published:** 2017-06-30

**Authors:** Go Shirota, Eriko Maeda, Yoko Namiki, Razibul Bari, Kenji Ino, Rumiko Torigoe, Osamu Abe

**Affiliations:** 1 Department of Radiology, Graduate School of Medicine, The University of Tokyo, 7-3-1 Hongo, Bunkyo-ku, Tokyo, 113-8655 Japan; 2 Imaging Center, The University of Tokyo Hospital, 7-3-1 Hongo, Bunkyo-ku, Tokyo, 113-8655 Japan; 3 Toshiba Medical Systems, 2-1-6, Tsukuda, Chuo-ku, Tokyo, 104-0051 Japan

**Keywords:** Children, Computed tomography, Congenital heart disease, Iterative reconstruction, Radiation dose

## Abstract

**Background:**

Full iterative reconstruction algorithm is available, but its diagnostic quality in pediatric cardiac CT is unknown.

**Objective:**

To compare the imaging quality of two algorithms, full and hybrid iterative reconstruction, in pediatric cardiac CT.

**Materials and methods:**

We included 49 children with congenital cardiac anomalies who underwent cardiac CT. We compared quality of images reconstructed using the two algorithms (full and hybrid iterative reconstruction) based on a 3-point scale for the delineation of the following anatomical structures: atrial septum, ventricular septum, right atrium, right ventricle, left atrium, left ventricle, main pulmonary artery, ascending aorta, aortic arch including the patent ductus arteriosus, descending aorta, right coronary artery and left main trunk. We evaluated beam-hardening artifacts from contrast-enhancement material using a 3-point scale, and we evaluated the overall image quality using a 5-point scale. We also compared image noise, signal-to-noise ratio and contrast-to-noise ratio between the algorithms.

**Results:**

The overall image quality was significantly higher with full iterative reconstruction than with hybrid iterative reconstruction (3.67±0.79 vs. 3.31±0.89, *P*=0.0072). The evaluation scores for most of the gross structures were higher with full iterative reconstruction than with hybrid iterative reconstruction. There was no significant difference between full and hybrid iterative reconstruction for the presence of beam-hardening artifacts. Image noise was significantly lower in full iterative reconstruction, while signal-to-noise ratio and contrast-to-noise ratio were significantly higher in full iterative reconstruction.

**Conclusion:**

The diagnostic quality was superior in images with cardiac CT reconstructed with electrocardiogram-gated full iterative reconstruction.

## Introduction

Cardiac CT is increasingly used for the diagnosis of infantile and pediatric heart disease [1–3]. Three-dimensional (3-D) isovolumetric datasets obtained from cardiac CT robustly assist morphological assessment by ultrasonography. However exposure to ionizing radiation remains a concern, particularly in infantile and pediatric populations.

Several technologies have been introduced to reduce the radiation dose in diagnostic imaging, such as prospective electrocardiogram-triggering [4–8] and iterative reconstruction methods, which enable good image quality at lower radiation exposure in pediatric CT [9–17]. Major CT vendors have developed hybrid iterative reconstruction techniques that are a combination of iterative reconstruction and filtered back-projection [18, 19]. Unlike hybrid iterative reconstruction, full iterative reconstruction techniques are based on both forward and backward projection. With backward projection steps, images are created using the projection data. Conversely, with forward projection steps projection data are created using the image data. The forward and backward projections are repeated until they do not change in subsequent iterations or until the maximum number of iterations is reached [15]. Full iterative reconstruction requires high computation power for image reconstruction, and this is one of the reasons it has taken until now for the development of full iterative reconstruction algorithms.

Three major CT vendors have recently introduced the following advanced model-based full iterative reconstruction algorithms: Veo (GE Healthcare, Waukesha, WI) and Iterative Model Reconstruction (IMR; Philips Healthcare, Best, The Netherlands) [15], and forward projected model-based iterative reconstruction solution (FIRST; Toshiba Medical Systems, Tokyo, Japan). Among these three full iterative reconstruction algorithms, FIRST is the only algorithm that can be combined with electrocardiogram-gated scan.

A previous study employing conventional radiation doses revealed that images obtained by the electrocardiogram-gated full iterative reconstruction method had less perceived image noise and better tissue contrast at similar resolution compared with an existing hybrid iterative reconstruction algorithm, namely Adaptive Iterative Dose Reduction 3D (AIDR 3D; Toshiba Medical Systems), which has been designed to be fully integrated into the automatic exposure control to reduce the radiation dose in pediatric cardiac CT [20]. However no study has evaluated the feasibility of this method in terms of the imaging quality for diagnosis of congenital heart disease and the ability to delineate cardiac anatomical structures in children.

Therefore the aim of this study was to compare the diagnostic quality of two reconstruction algorithms, full iterative reconstruction and hybrid iterative reconstruction, in low-dose 320-row pediatric cardiac CT, particularly in terms of delineation of cardiac anatomical structures of children.

## Materials and methods

### Patients

We obtained parental written informed consent for contrast-enhanced CT in all children. The local ethics committee approved the study protocol and waived the requirement for informed consent to retrospectively review the CT examinations.

We retrospectively examined CT images of children with congenital heart disease requiring surgical or catheter intervention and without renal dysfunction (effective glomerular filtration rate<40 mL/min) who underwent cardiac CT from September 2015 to March 2016. We included CT images of 49 children (ages 5 days to 5 years 10 months, median 122 days; 25 males and 24 females; body weight 2.5–28.0 kg, median 5.0 kg).

In total, 43/49 children had complex (≥2) congenital heart diseases, while 6 had a single disease. Types of congenital heart disease are shown in Table 1. Twenty-four examinations were performed before surgical or catheter intervention, while 25 examinations were performed after ≥1 surgical or catheter intervention. Procedure types performed prior to examinations are shown in Table 2. One child had an implanted pacemaker before CT scan; however because the generator was implanted in the abdominal wall, the metal artifact from the generator and the pacemaker lead was negligible in our image analysis.

**Table 1:** Frequency of defects in 49 children with congenital heart disease

Defect	Number of children
Atrial septal defect	32
Ventricular septal defect	28
Patent ductus arteriosus	19
Pulmonary artery stenosis	17
Double-outlet of the right ventricle	9
Pulmonary atresia	8
Single ventricle	7
Single atrium	7
Transposition of the great arteries	6
Hypoplastic left heart syndrome	6
Tetralogy of Fallot	5
Tricuspid atresia	4
Total anomalous pulmonary venous return	3
Coarctation of the aorta	3
Hypoplasia of right ventricle	3
Truncus arteriosus communis	2
Pulmonary venous obstruction	1

Of the 49 children enrolled in this study, 43 had two or more defects

**Table 2:** Types of procedures performed prior to CT

Procedure	Number of children
Pulmonary artery banding	10
Pulmonary angioplasty	10
Blalock–Taussig shunt	7
Norwood procedure	5
Glenn procedure	5
Ventricular septal defect closure	3
Atrial septal defect closure	1
Fontan procedure	1
Aorta to pulmonary artery shunting	1
Pulmonary venous stenting	1
Unifocalization of major aortopulmonary collaterals	1
Balloon atrioseptostomy	1
Aortic coarctation repair	1
Pacemaker implantation^a^	1

A total of 24 examinations were performed before surgical or catheter intervention, while 25 examinations were performed after one or more surgical or catheter interventions

^a^ The generator was implanted in the abdominal wall so the metal artifact from the generator and the pacemaker lead was negligible in our image analysis

### CT data acquisition and image reconstruction

All children underwent angiography via second-generation 320-row CT (Aquilion ONE ViSION edition; Toshiba Medical Systems, Tochigi, Japan) with electrocardiogram-gated axial scans. Scan parameters were as follows: tube potential, 80 kVp; gantry rotation time, 275 ms; and tube current determined by auto exposure control (a predetermined level of image noise set at a standard deviation of 40).

Children in the study received contrast enhancement material at 2 mL/kg body weight of 300 mgI/mL of iohexol (Omnipaque 300, 300 mg/mL; Daiichi Sankyo, Tokyo, Japan).

For children <6 months or with a body weight <5 kg, the contrast-enhancement material was diluted by adding normal saline at one-half volume of the material and injected at a rate of 0.5 mL/s. For patients >6 months or with a body weight of >5 kg, undiluted contrast-enhancement material was injected at a rate of 1.0 mL/s.

For each child, an experienced cardiovascular radiologist and senior technologist determined the phase with minimum artifacts at the CT console. Multiple phases were reconstructed if image artifacts persisted. The slice thickness of reconstructed images was 0.50 mm with increments of 0.25 mm. Images were reconstructed using two algorithms: a medium soft-tissue kernel (FC04) with a hybrid iterative reconstruction algorithm (AIDR 3D enhanced strong mode with ^SURE^Exposure) and full iterative reconstruction (FIRST in the “cardiac strong” mode).

### Radiation dose

Radiation dose exposure was assessed as the volume CT dose index (CTDI_vol_, mGy) and dose–length product (mGy·cm). With the dose–length product displayed by the CT system after the examination (phantom size of 32 cm), the effective dose, *E*, for each child was calculated as follows: E=k x dose–length product, where *k* is the conversion coefficient for chest CT at 80 kV with values of 0.0823, 0.0525, 0.0344 and 0.248 for newborns, children age <1, age 1–4 and age 5–10 years, respectively, based on a previous report [21]. Size-specific dose estimates (SSDEs) were also calculated. Anterior-posterior and lateral diameters were measured on transverse CT images at the level of the aortic valve. For the sum of these diameters in each child, conversion factors were chosen from the table of the report of AAPM task group 204 for a phantom size of 32 cm [22]. SSDEs were calculated as the CT dose index × conversion factor (mGy).

### Subjective image analysis

Subjective image quality was rated by two cardiovascular radiologists (G.S. and E.M., with 7 years and 15 years of experience in pediatric and cardiovascular radiology, respectively), who were blinded to the details of the CT datasets, which were provided in a randomized order.

A 3-point scale (3=diagnostic/2=diagnostic with limitations/1=non-diagnostic) was used to score the delineation of the following anatomical structures: atrial septum, ventricular septum, right atrium, right ventricle, left atrium (including the pulmonary veins), left ventricle, main pulmonary artery, aortic arch (including the patent ductus arteriosus), descending aorta and coronary arteries (right coronary artery and left main trunk).

Artifacts from medical devices (e.g., electrodes and cables for electrocardiography) were not evaluated. Before the scan, metallic items were positioned as far as possible from the scan range.

**Fig. 1:**
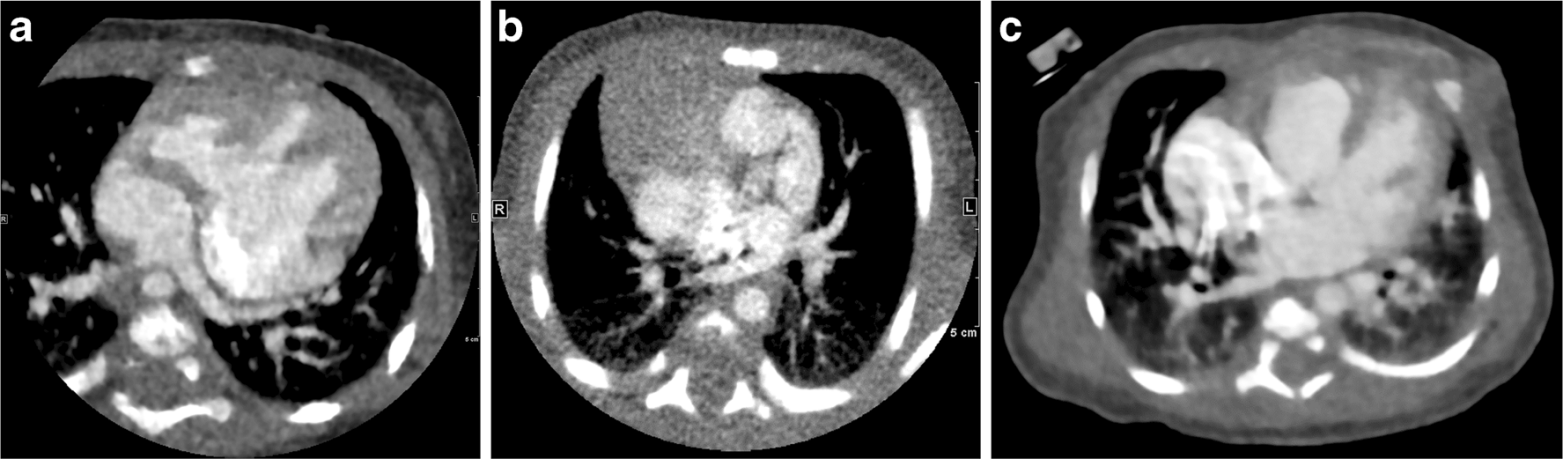
Representative axial contrast-enhanced CT images for the three levels of beam-hardening artifacts. **a** Score 3, no beam-hardening artifact, in a 1-month-old boy with single atrium, single ventricle, triscupid atresia and double-outlet of the right ventricle. **b** Score 2, mild artifact, defined as images that still allow evaluation of the surrounding anatomical structures. This image is in a 7-day-old boy with single atrium, single ventricle, pulmonary artery stenosis and total anomalous pulmonary venous return. **c** Score 1, severe artifacts that preclude evaluation, in a 10-day-old girl with atrial septal defects and ventricular septal defect

A 3-point scale was used to evaluate the presence of beam-hardening artifact from contrast-enhancement material: 3=no artifact, 2=mild artifact that still allowed evaluation of the (surrounding) anatomical structures, and 1=severe artifact that precluded evaluation. Representative images for three levels of beam-hardening artifact are shown in Fig. 1.

**Fig. 2:**
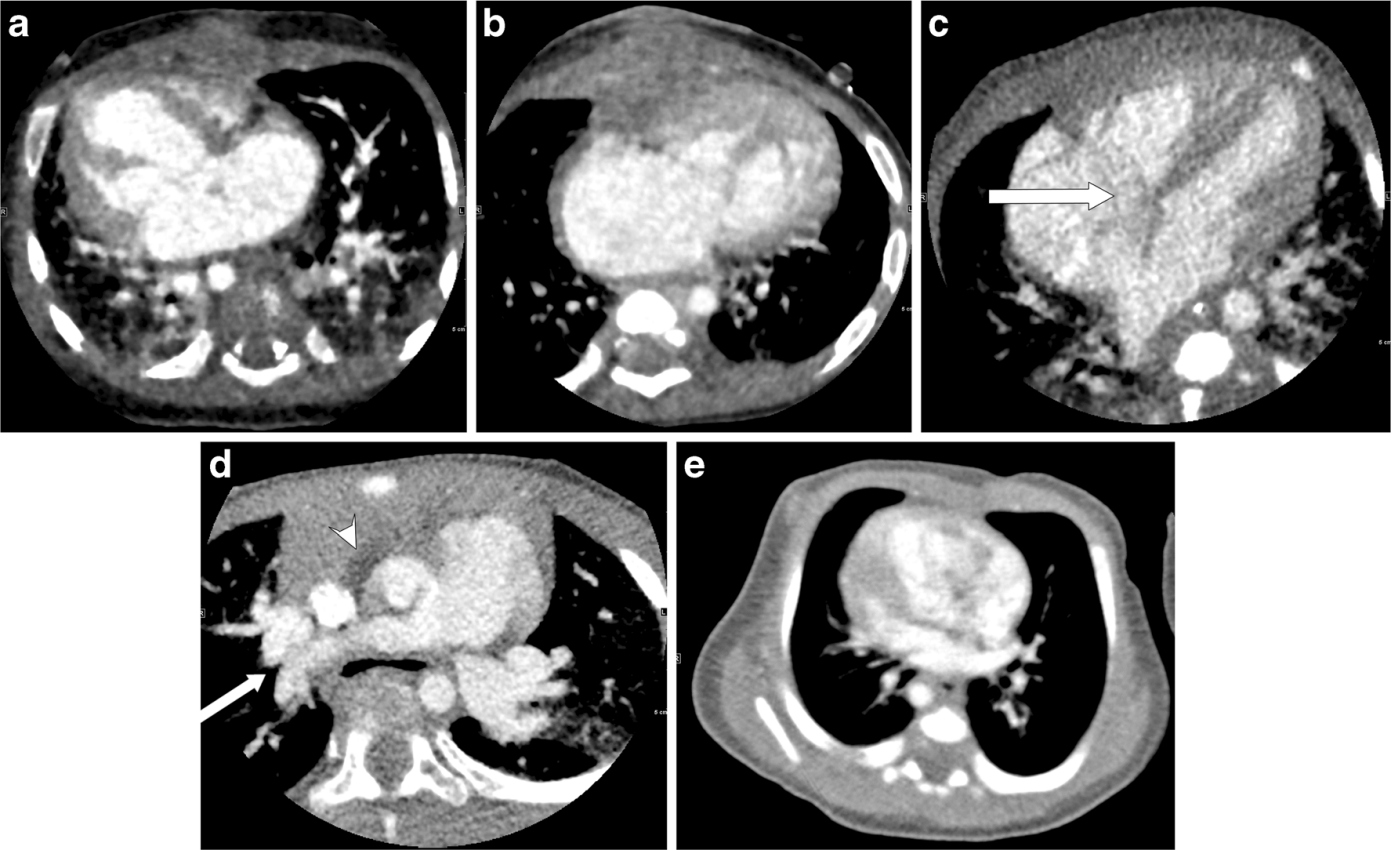
Representative axial contrast-enhanced CT images of the five levels of overall image quality. **a** Score 5, excellent anatomical clarity and image quality, in a 2-month-old girl with atrial septal defects and ventricular septal defect. **b** Score 4, good anatomical clarity and image quality with minor motion artifacts, in a 6-month-old boy with hypoplastic left heart syndrome after Norwood procedure and pulmonary angioplasty. **c** Score 3, fair image quality with motion artifacts extending less than 5 mm from the vessel center *arrow*, here in a 5-day-old boy with transposition of the great arteries, atrial septal defect and patent ductus arteriosus. **d** Score 2, poor image quality, inadequate delineation between the vessel and surrounding tissue *arrow*, presence of streak artifacts extending at least 5 mm from the center of the vessel *arrowhead*, and no useful information obtained. This image is in a 2-year-old girl with atrial septal defect. **e** Score 1, non-diagnostic image quality, in a 1-month-old girl with transposition of the great arteries

The overall image quality was evaluated using the following 5-point scale: 5=excellent anatomical clarity and image quality; 4=good anatomical clarity and image quality with minor motion artifact; 3=fair image quality with motion artifact extending <5 mm from the vessel center; 2=poor image quality (inadequate delineation between the vessel and surrounding tissue, presence of streak artifacts extending at least 5 mm from the center of the vessel, and no useful information obtained); and 1=non-diagnostic image quality. Representative images of five levels of the overall image quality are shown in Fig. 2.

Subjective image quality was defined as diagnostic when the scores were equal to or greater than 2 on the 3-point scale and equal to or greater than 3 on the 5-point scale. The assessment scale for image quality was based on a previous pediatric study using cardiovascular CT angiography [23].

### Objective image analysis

For objective image analysis, we calculated image noise, signal-to-noise ratio (SNR) and contrast-to-noise ratio (CNR) according to the method proposed by Pflederer et al. [24]. Regions of interest were defined on an axial image at the level of the proximal ascending aorta. The average CT number and noise were recorded using a circular region of interest. The region of interest was made as large as possible while carefully avoiding inclusion of the vessel wall to prevent partial volume effects. A region of interest was placed immediately next to the vessel contour on an axial image and the average CT number was recorded. Image noise was defined as the average standard deviation of the circular region of interest placed at the ascending aorta. Signal-to-noise ratio was defined as ratio of CT number of ascending aorta divided by the image noise. Contrast-to-noise ratio was calculated as the difference in the CT number between the ascending aortic lumen and nearby connective tissue divided by the image noise. Image noise, SNR and CNR were calculated for both full iterative reconstruction and hybrid iterative reconstruction by a cardiovascular radiologist (G.S., with 7 years of experience in pediatric and cardiovascular radiology) who was blinded to the details of the CT datasets. These indicators were compared between the two algorithms.

### Statistical analysis

We compared pairs of subjective evaluation scores and objective image-quality indicators for full iterative reconstruction and hybrid iterative reconstruction for each patient. Scores were presented as means ± standard deviations using the Wilcoxon signed-rank test. We calculated interobserver agreement for subjective image quality using Cohen’s k statistic [25] and interpreted it as poor (k<0.20), fair (k=0.21–0.40), moderate (k=0.41–0.60), good (k=0.61–0.80), very good (k=0.81–0.90) or excellent (k≥0.91). We conducted all statistical analyses using JMP software (version 12.0.0; SAS Institute, Cary, NC). *P<*0.05 was statistically significant.

## Results

### Radiation exposure

Indicators of radiation exposure are summarized in Table 3. The average tube current was 52.7±15.6 mA. A low radiation dose was achieved by the proposed protocol, as indicated by the average effective dose of 0.37±0.23 mSv and average SSDE of 1.36±0.69 mGy.

**Table 3:** Summary of radiation exposures

Variable	Overall	Newborn	<1 year	<5 years
Tube current (mA)	52.7±15.6 (range 30–90)	40.0±8.7 (range 30–60)	50.8±11.6 (range 35–80)	70.0±12.3 (range 50–90)
CTDI_vol_ (mGy)	0.56±0.29 (range 0.2–1.7)	0.45±0.39 (range 0.2–1.7)	0.52±0.19 (range 0.3–1.2)	0.78±0.27 (range 0.4–1.4)
Dose–length product (mGy·cm)	7.54±4.64 (range 2.2–22.4)	4.78±4.20 (range 2.2–17.0)	6.96±3.48 (range 3.7–19.5)	11.96±4.86 (range 5.3–22.4)
Effective dose (mSv)	0.37±0.23 (range 0.18–1.40)	0.39±0.35 (range 0.18–1.40)	0.37±0.18 (range 0.19–1.02)	0.41±0.17 (range 0.18–0.77)
SSDE (mGy)	1.36±0.69 (range 0.54–4.57)	1.18±1.05 (range 0.54–4.57)	1.28±0.46 (range 0.81–3.00)	1.73±0.56 (range 0.96–3.02)

*CTDI*_*vol*_ volume CT dose index, *SSDE* size-specific dose estimate

### Subjective image quality

Evaluation of subjective image quality is summarized in Table 4. Interobserver agreement for image quality according to the Cohen’s k statistic was very good or excellent.

**Table 4:** Summary of subjective image quality

Landmark	n^a^	Rating scale	Weighted kappa	Full iterative reconstruction (mean±SD)	Hybrid iterative reconstruction (mean±SD)	*P*-value^b^
A-septum	46	1–3	0.858	2.30±0.58	2.13±0.46	0.0099
V-septum	48	1–3	0.915	2.70±0.41	2.60±0.45	0.1098
Right atrium	48	1–3	0.892	2.22±0.49	2.18±0.44	0.4783
Right ventricle	48	1–3	0.925	2.60±0.41	2.54±0.47	0.2755
Left atrium^c^	49	1–3	0.926	2.68±0.40	2.49±0.52	0.0038
Left ventricle	49	1–3	0.913	2.79±0.31	2.62±0.45	0.0060
mPA	39	1–3	0.873	2.53±0.43	2.40±0.46	0.1298
Ascending aorta	49	1–3	0.908	2.64±0.42	2.60±0.44	0.2862
Arch/PDA	49	1–3	0.929	2.68±0.48	2.72±0.37	0.7468
Descending aorta	49	1–3	0.980	2.94±0.22	2.88±0.28	0.2047
RCA	49	1–3	0.921	2.18±0.81	2.01±0.74	0.0048
LMT	49	1–3	0.912	2.22±0.78	2.05±0.69	0.0035
CM beam hard	49	1–3	0.913	2.35±0.58	2.24±0.60	0.1779
Overall	49	1–5	0.963	3.67±0.79	3.31±0.89	0.0072

*A-septum* atrial septum, *Arch/PDA* aortic arch including the patent ductus arteriosus, *CM beam hard* beam-hardening artifacts from contrast material, *LMT* left main trunk, *mPA* main pulmonary artery, *RCA* right coronary artery, *SD* standard deviation, *V-septum* ventricular septum

^a^Some cases were excluded when the relevant structure did not exist congenitally

^b^Wilcoxon signed-rank test; *P*<0.05 was considered significant

^c^Left atrium including the pulmonary veins

**Fig. 3:**
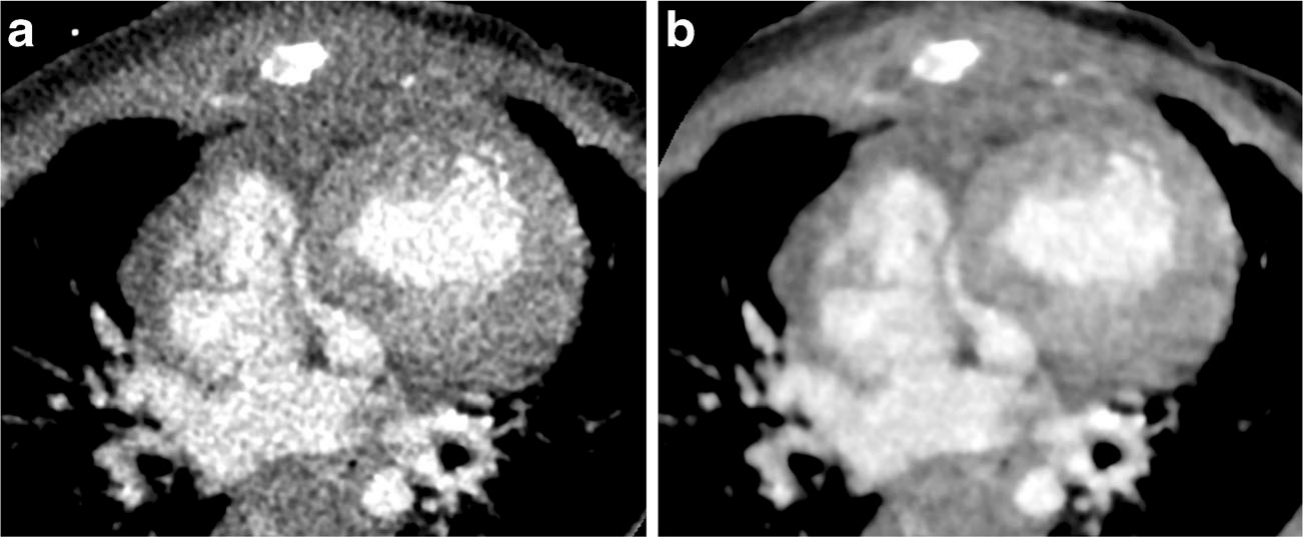
Axial contrast-enhanced CT images in a 4-month-old boy with hypoplastic left heart syndrome. **a** Hybrid iterative reconstruction. **b** Full iterative reconstruction. Noise, graininess, and contrast resolution between cardiac and coronary structures and the lumen are significantly improved with full iterative reconstruction as compared with hybrid iterative reconstruction. The CT window settings of both figures are identical

**Fig. 4:**
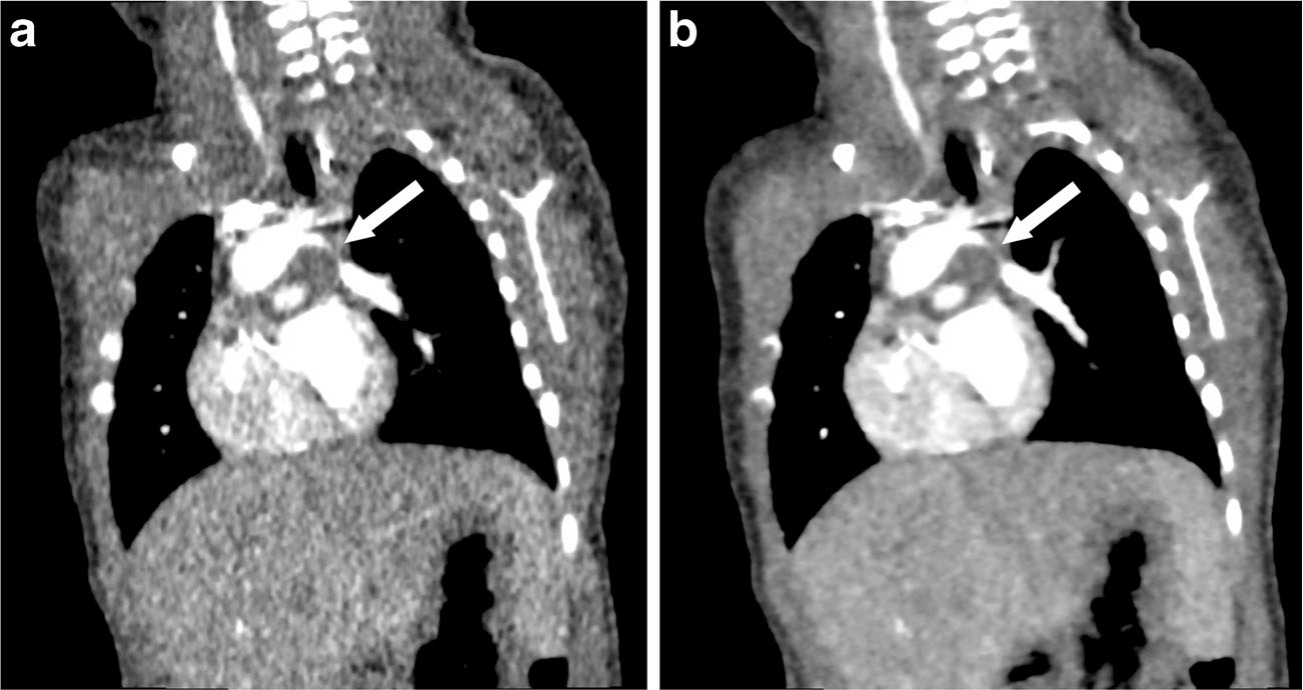
Coronal contrast-enhanced CT images in a 7-day-old boy with a double-outlet right ventricle, aortic origin of the left pulmonary artery, severe left pulmonary artery stenosis, ventricular septal defect and atrial septal defect. **a** Hybrid iterative reconstruction. **b** Full iterative reconstruction. Although left pulmonary artery stenosis (*arrow*) is brokenly depicted with hybrid iterative reconstruction and patency is ambiguous, patency is better demonstrated with full iterative reconstruction. The CT window settings of both figures are identical

**Fig. 5:**
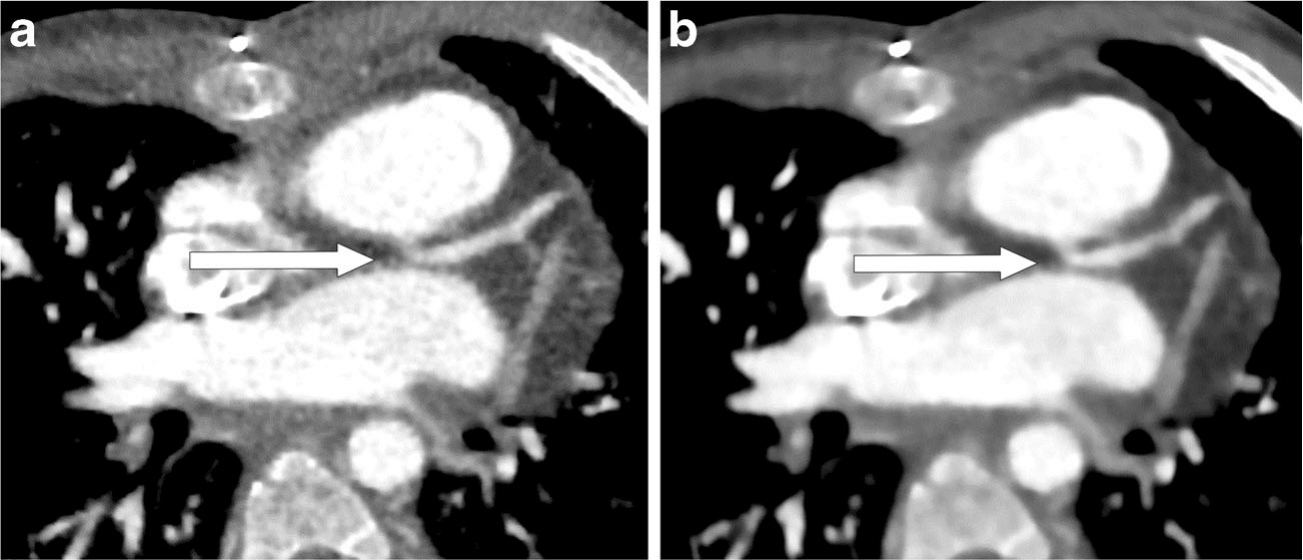
Axial contrast-enhanced CT images in a 5-year-old girl with truncus arteriosus. **a** Hybrid iterative reconstruction. **b** Full iterative reconstruction. The left main trunk originating from a noncoronary cusp (*arrow*) is demonstrated more clearly with full iterative reconstruction than with hybrid iterative reconstruction. The CT window settings of images in both figures are identical

For both algorithms, the scores for each structure and beam-hardening artifact were greater than 2/3 and the overall quality was greater than 3/5, which means that subjective image quality was diagnostic. For most gross structures, the evaluation scores were higher with full iterative reconstruction than with hybrid iterative reconstruction. There were significant differences in the scores for the atrial septum, left atrium, left ventricle, right coronary artery and left main trunk. Overall scores were significantly higher with full iterative reconstruction than with hybrid iterative reconstruction. There was no significant difference between full iterative reconstruction and hybrid iterative reconstruction with respect to the presence of beam-hardening artifacts from the contrast-enhancement material. Representative images reconstructed with both algorithms are shown in Figs. 3, 4 and 5.

### Objective image quality

Evaluation of objective image quality is summarized in Table 5. Image noise was significantly lower in full iterative reconstruction, while SNR and CNR were significantly higher in full iterative reconstruction.

**Table 5:** Summary of objective image quality

	Full iterative reconstruction (mean±SD)	Hybrid iterative reconstruction (mean±SD)	*P*-value^a^
Image noise (HU)	28.8±10.7	49.7±11.4	<0.0001
SNR	15.4±5.5	9.3±2.7	<0.0001
CNR	13.9±5.7	8.5±3.1	<0.0001

*CNR* contrast-to-noise ratio, *HU* Hounsfield units, *SD* standard deviation, *SNR* signal-to-noise ratio

^a^Wilcoxon signed-rank test. *P<*0.05 was statistically significant

## Discussion

Because of increasing concern about exposure to ionizing radiation, it is necessary to develop low-dose cardiac CT scanning procedures, particularly for use in children.

Unlike hybrid iterative reconstruction, wherein noise is independently reduced in sinograms and image spaces, full iterative reconstruction has fewer streak artifacts and improved spatial resolution on sinograms through forward projection jointly using data from the fidelity, optic, system, cone-beam and statistical noise models. Three major CT vendors have recently introduced advanced model-based full iterative reconstruction algorithms: Veo (GE Healthcare), Iterative Model Reconstruction (IMR; Philips Healthcare), and forward projected model-based iterative reconstruction solution (FIRST; Toshiba Medical Systems). Among these three full iterative reconstruction algorithms, FIRST is the only algorithm that can be combined with electrocardiogram-gated scan. Another feature of FIRST compared to other iterative reconstruction algorithms (Veo and IMR) is its regularization process. Using forward-projected data, further noise reduction is achieved using an anatomical model via the regularization process and adaptive iteration. The regularization process is optimized for specific organs (e.g., bone, heart, lung and abdomen) to reduce image noise. This series of forward-projection and regularization processes results in improved noise reduction, spatial resolution and density resolution compared with hybrid iterative reconstruction.

The results of this study revealed that low-dose CT with a 320-row detector and full iterative reconstruction has good diagnostic quality for gross structures. The present 320-row cardiac CT achieved dose levels close to those of the diagnostic reference level for pediatric chest radiographs. Internationally reported effective doses for conventional chest radiography range from 0.01 mSv to 0.299 mSv [26, 27]. Moreover, for most structures the evaluation scores with full iterative reconstruction were higher than those with hybrid iterative reconstruction, particularly for the atrial septum, left atrium, left ventricle, right coronary artery and left main trunk. As shown in Fig. 1, contrast resolution between cardiac and coronary structures and the lumen were significantly improved with full iterative reconstruction over hybrid iterative reconstruction, in accordance with the hypothesis of this study. According to a previous study that compared image quality between full iterative reconstruction and hybrid iterative reconstruction in adult cardiac CT, the SNR was significantly higher with full iterative reconstruction [28]. The result of objective image analysis in the present study revealed that image noise was significantly higher in hybrid iterative reconstruction, while SNR and CNR were significantly higher in full iterative reconstruction. Further experimental studies are necessary to prove this hypothesis.

For evaluation of image quality for left atrium and left ventricle there was a significant difference between full iterative reconstruction and hybrid iterative reconstruction, whereas there was no significant difference for the right atrium and right ventricle. For the right atrium, particularly in the early phase, difficulty existed in evaluation with both algorithms because of turbulent flow caused by the mixture of highly concentrated contrast-enhancement material. For the right ventricle, the contrast material was inhomogeneously distributed because of thick trabeculae and less motion; therefore the right ventricle was difficult to evaluate using both algorithms.

There were several limitations to this study. The diagnostic accuracies of hybrid iterative reconstruction and full iterative reconstruction were not assessed using conventional cardiac angiography. Second only the “cardiac” mode was applied. However the feasibility of the “cardiac-sharp” mode should be validated in further studies. Another limitation is on blindness of subjective image analysis. Because of the study design for the subjective image analysis, recognizing the algorithm seemed to be possible for the readers and this raises a doubt on the validity of the results.

## Conclusion

Compared with hybrid iterative reconstruction, full iterative reconstruction provides better depiction with 320-row pediatric cardiac CT. Image quality of low-dose cardiac CT reconstructed with electrocardiogram-gated model-based full iterative reconstruction is clinically acceptable for children.
